# An overview of HCV molecular biology, replication and immune responses

**DOI:** 10.1186/1743-422X-8-161

**Published:** 2011-04-11

**Authors:** Usman A Ashfaq, Tariq Javed, Sidra Rehman, Zafar Nawaz, Sheikh Riazuddin

**Affiliations:** 1 Division of Molecular Medicine, National Centre of Excellence in Molecular Biology, University of the Punjab, Lahore, Pakistan; 2 Braman Family Breast Cancer Institute, University of Miami, USA; 3 Allama Iqbal Medical College, University of Health sciences, Lahore

**Keywords:** HCV, replication, HCV entry, immune responses

## Abstract

Hepatitis C virus (HCV) causes acute and chronic hepatitis which can eventually lead to permanent liver damage, hepatocellular carcinoma and death. Currently, there is no vaccine available for prevention of HCV infection due to high degree of strain variation. The current treatment of care, Pegylated interferon α in combination with ribavirin is costly, has significant side effects and fails to cure about half of all infections. In this review, we summarize molecular virology, replication and immune responses against HCV and discussed how HCV escape from adaptive and humoral immune responses. This advance knowledge will be helpful for development of vaccine against HCV and discovery of new medicines both from synthetic chemistry and natural sources.

## Background

Hepatitis C virus (HCV) infection is a serious global health problem that affects 180 million people worldwide and 10 million people in Pakistan [1]. It is estimated that three to four million people are infected with HCV every year [2]. Hepatitis C virus (HCV) causes acute and chronic hepatitis which can eventually lead to permanent liver damage and hepatocellular carcinoma [2]. Of those acutely infected with HCV, around 85% develop chronic infection. Approximately 70% of patients with chronic viremia develop chronic liver disease, 10-20% of which develop liver cirrhosis. Hundreds of thousands of people die each year from liver failure and liver cancer caused by this disease.

**Figure 1:**
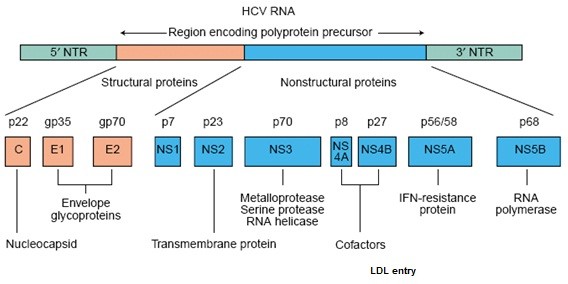
**Proteins encoded by the HCV genome**. HCV is formed by an enveloped particle harbouring a plus-strand RNA of 9.6 kb. The genome carries a long openreading frame (ORF) encoding a polyprotein precursor of 3010 amino acids. Translation of the HCV ORF is directed via a 340 nucleotide long 5' nontranslated region (NTR) functioning as an internal ribosome entry site; it permits the direct binding of ribosomes in close proximity to the start codon of the ORF. The HCV polyprotein is cleaved co- and post-translationally by cellular and viral proteases into ten different products, with the structural proteins (core (C), E1 and E2) located in the N-terminal third and the nonstructural (NS2-5) replicative proteins in the remainder. Putative functions of the cleavage products are shown [4].

HCV is a small enveloped virus with a positive-sense, single-stranded RNA genome that encodes a large polyprotein of 3010 amino acids. The polyprotein is co- and posttranslationally processed by cellular and virally encoded proteases to produce the mature structural and non-structural (NS) proteins. Among the NS proteins, the NS3 serine-like protease and the RNA-dependent RNA polymerase (RdRp) are essential for viral maturation and replication, and therefore represent ideal targets for the development of small molecule anti-HCV compounds (Figure 1) [3,4].

## HCV Structural Proteins

### Core protein

HCV core is a highly conserved basic protein which makes up the viral nucleocapsid. Core consists of HCV first 191 amino acids and can be divided into three domains on the basis of hydrophobicity. Domain 1 (amino acids 1 - 117) contains mainly basic residues with two short hydrophobic regions. Domain 2 (amino acids 118 - 174) is less basic and more hydrophobic and its C -terminus is at the end of p21. Domain 3 (amino acids 175 - 191) is highly hydrophobic and acts as a signal sequence for E1 envelope protein [5]. Core protein can bind viral RNA [6] via domain 1 (amino acids 1 - 74). Core is a cytosolic membrane-bound protein, which has been found to associate with the endoplasmic reticulum (ER), lipid droplets, mitochondria and the nucleus. Core protein directly or indirectly involved in hepatocarcinogenesis and steatosis hepatitis [7,8]. HCV core protein interacts with numerous cellular proteins and to affect host cell functions such as gene transcription, lipid metabolism, apoptosis and various signaling pathways [9]

### Envelope glycoproteins

HCV consist of two "envelop proteins" E1 and E2 (Figure 1). These proteins are highly glycosylated and play an important role in cell entry. E1 serves as the fusogenic subunit and that E2 acts as the receptor binding subunit of the HCV envelope. The E1 envelope glycoprotein of HCV contains 4 to 5 N-linked glycans and the E2 envelope glycoprotein has 11 N-glycosylation sites [10,11]. However the numbers of glycosylation sites vary according to genotype. Glycosylation sites on E1 and E2 are highly conserved and contain a mixture of complex and high-mannose side-chains. HCV glycans play an important role in envelope glycoprotein folding and formation of the HCV E1E2 complexes, receptor interactions with virus. [11] and antigenic variation [12].

The envelope proteins are thought to mediate cell entry by recognition of cellular membrane receptor proteins. However, until recently, research in this area was difficult due to the lack of infectious cell based systems. The development of cells, which produce infectious HCV pseudotype particles (HCVpp) by the use of a retroviral vector for assembly of the virus pseudoparticle has helped the identification of cellular receptors [13]. Furthermore, HCVpp could be neutralised by anti-E2 monoclonal antibodies [14]

Various putative cellular receptors have been suggested as mediating interactions with HCV envelope proteins. Truncated forms of E2 have been shown to interact with CD81, scavenger receptor type B class 1 protein (SRB-1) and high density lipoprotein (HDL) binding molecule [15,16]. Soluble forms of CD81 can inhibit entry of HCVpp to cells [14]. Ectopic expression of CD81 in CD81-negative cells does not permit HCVpp entry indicating that CD81 is a co-receptor. Another proposed HCV receptor is the low density lipoprotein (LDL) receptor, which was shown to help endocytosis of the virus. Viral entry could be prevented in a number of cell types using an anti-LDL monoclonal antibody [17]. Mannose binding proteins (DC-SIGN and L-SIGN) have been suggested as to have interactions with E2 but their contribution to viral entry is not known [18].

E2 contains two hypervariable regions (HVR), HVR1 and HVR2, which are under constant selection for mutation probably because they are targets for neutralizing antibodies. Numerous studies have highlighted the genetic heterogeneity of the HVR1, which may enable virus to evade the immune system and facilitate establishment of chronic infection [19,20]. However, chronic infection has been reported in an experimentally infected chimpanzee even though there was no variation in HVR [21].

### P7 Protein

P7 is a 63-amino acid polypeptide located between HCV E2 and NS2 genes. P7 is a membrane-spanning protein located in the Endoplasmic Reticulum (ER). The cleavage of p7 is mediated by the ER signal peptidases of the host cell. Two transmembrane domains (TMDs) of P7 are connected by a cytoplasmic loop and oriented towards the ER lumen. It also has been demonstrated that the carboxyl-terminal TMD of P7 can function as a signal sequence that most likely promotes the translocation of NS2 into the ER lumen for appropriate cleavage by host signal peptidases. These proteins form ion channels that play an essential role in virus infection [22]. HCV P7 has characteristics similar to those of a group of proteins called viroporins. P7 has been shown to be essential for virus particle assembly and release of infectious virions in a genotype specific manner [23].

## Nonstructural proteins

### NS2

NS2 protein is a 21-23 kDa transmembrane protein. NS2 protein is essential for completion of the viral replication cycle *in vitro *and *in vivo *[24,25]. NS2 contains highly hydrophobic N-terminal residues forming three or four transmembrane helices that insert into the ER membrane. The C-terminal part of NS2 presumably resides in the cytoplasm play an important role in NS2/3 auto protease activity together with the N-terminal domain of NS3. The domain required for this cleavage was mapped between amino acids 827 and 1207 of the polyprotein at the C-terminus of NS2 [26]. NS2-3 was called a metalloprotease based on observations that exogenous zinc stimulated protease activity and chelating agents, like EDTA, were able to inhibit protease activity. The zinc, which is known to be essential, may act structurally to stabilise the NS3 structure at the active site. Analysis of the region showed that amino acid requirements for effective NS2-3 cleavage vary between HCV strains but deletion was required in both NS2 and NS3 to inhibit cleavage [27]. The crystal structure of the C-terminal domain of NS2 has recently been determined and reveals a dimeric protease containing two composite active sites [28].

### NS3

The NS3 is 67 kDa protein with multifunctional activity. NS3 N-terminal has serine protease activity and a C-terminal has NTPase/helicase activity [29]. NS3 protein bound with ER membrane along with NS4A protein [30]. HCV NS3 protease last 185 amino acids at the N-terminus involved in cleavage between NS3-4A, 4A-4B, 4B-5A and 5A-5B [31]. The proposed catalytic activity of HCV NS3 is due to three amino acid residues His- 1083, Asp-1107 and Ser-1165. Replacement of His-1083 and Ser-1165 with alanine abolished NS3 cleavage of the HCV polyprotein without affecting protein structure of NS3 [32,33]. The NS3 protein also contained a short consensus sequence, which interacted with the catalytic subunit of protein kinase A (PKA). This interaction led to retention of the catalytic subunit of PKA in the cytoplasm preventing it entering the nucleus. PKA modifies intracellular proteins by adding phosphate groups altering target protein function. Therefore, NS3/PKA interactions may deregulate intracellular signalling [34]. NS3 serine protease recently turned out to influence the innate cellular host defense by inhibition of RIG-I and TLR3 signalling [35]. The NTPase/helicase domain of NS3 resides in the C-terminal 465 residues of the NS3 protein.

The enzymatic activity of the NS3 NTPase/helicase activity is indispensable for RNA replication. Putative functions during replication could be to unwind replicative double stranded RNA intermediates, to eliminate RNA secondary structures or to separate the genome from nucleic acid binding proteins. Recent advances in the understanding of the molecular mechanisms of this enzyme could enable a specific inhibition as a novel antiviral strategy [36,37].

### NS4A

NS4A is a 54 amino acids protein, which acts as a cofactor for NS3 protein. The NS4A protein has an N-terminus which is highly hydrophobic and deletion analysis showed it to be involved in targeting NS3 to the ER membrane [30]. It was proposed that last 20 amino acids form a transmembrane helix, which anchors the NS3/NS4A complex on the ER membrane. The interaction between NS4A and NS3 is mediated between residues within the core of NS3 and the C- terminus of NS4A. This interaction allows activation of the NS3 active site and more efficient protease cleavage [38]. NS4A is also required for the phosphorylation of NS5A and can directly interact with NS5A. Deletion analysis indicated that a region of amino acids in the centre of NS5A (amino acids 2135 to 2139) was essential for NS4A-dependent phosphorylation of NS5A [39].

### NS4B

NS4B is a small hydrophobic 27 kDa protein, which play an important role for recruitment of other viral proteins. Topology studies have found NS4B contains 4 transmembrane domains. The cytoplasmic C-terminus of NS4B and the N-terminus has a dual topology where most faces the ER lumen [40]. NS4B interacts with NS4A and therefore indirectly with NS3 and NS5A [41]. The NS4B protein was found to be an integral membrane protein which was targeted to the ER and co-localized with other non-structural proteins in the ER membrane [42]. Electron microscopy studies indicated that NS4B induced morphological changes to the ER forming a structure termed the membranous web. All viral proteins were localized to this area suggesting a site for replication complex formation [43]. Additional immunofluorescence studies indicated that NS4B has reduced mobility in these foci which may be due to oligomerisation [44]. NS4B protein failed to show cytopathic or oncogenic effects in the livers of transgenic mice [45].

### NS5A

NS5A is a hydrophilic phosphoprotein which plays an important role in viral replication, modulation of cell signaling pathways and interferon response [46,47]. NS5A contains no transmembrane domains. The membrane association of NS5A is mediated by a unique amphipathic alpha helix which is localized at the N-terminus [48,49]. NS5A play an important role in replication. Initial studies indicated the association of NS5A with other viral proteins which suggested its presence in replication complexes [50]. Mutation of the amphipatic helix disrupted membrane association and prevented formation of replicon-harbouring cells [51]. Mutations in NS5A were essential for viral replication and establishing a replicon cell line [52]. NS5A associated with lipid droplets when expressed alone or as part of the polyprotein [53]. Structural analysis of the N-terminus showed NS5A contained an essential zinc-coordination motif, which play an important in structural integrity [54].

NS5A has initially attracted considerable interest because of its potential role in modulating the IFN response [55]. NS5A contain a region, which confers resistance of the virus to interferon treatment [56]. This region, called the interferon-α sensitivity-determining region (ISDR), was later found to interact directly with an IFN-α stimulated gene product, PKR protein kinase. PKR protein kinase is activated by binding to double-stranded RNA resulting eventually in cessation of protein synthesis. It was proposed that sequences in the ISDR could be used to predict sensitivity or resistance of HCV to IFN-α treatment [57].

NS5A has been proposed as having numerous interactions with proteins affecting cell signalling. NS5A can modulate the three main MAPK pathways involved in host cell mitogenic signalling, which regulate growth and activation. NS5A is able to regulate cellular signalling by both pro- and anti- apoptotic mechanisms. It has also been implicated in interfering with ROS pathways and phosphatidylinositol 3-kinase signalling pathways, which may lead to hepatocyte transformation and HCC formation [46].

### NS5B

The NS5B is a tail anchored protein of 65 kDa in size. NS5B acts as RNA dependent RNA polymerase and plays an important role in synthesis of new RNA genome [58]. Sequence analysis had identified an amino acid motif GDD play an essential role for polymerase activity [59]. The structural organization of NS5B is a typical 'right hand' polymerase shape with finger, palm, and thumb sub domains surrounding a completely encircled active site [60]. Replication proceeds *via *synthesis of a complementary minus-strand RNA using the genome as a template and the subsequent synthesis of genomic plus-strand RNA from this minus-strand RNA intermediate. As central component of the HCV replicase, NS5B has emerged as a major target for antiviral intervention [61].

## HCV virology

On the basis of nucleotide variation HCV is divided into six major genotypes and more than 80 subtypes. The highest sequence variability concentrated in hypervariable region of E1 and E2 glycoprotein. The lowest sequence variability between genotypes is found in the 5' untraslated region (UTR) which contain specific sequences and RNA secondary structures that are required for replication and translation functions. The sequence variability is due to high replication rate and lack of proofreading activity of RNA-dependent RNA polymerase. The rate of nucleotide misincorporation is approximately 10^-3 ^base substitutions per genome site per year [62]. HCV infection is a highly dynamic process with a viral half-life of only a few hours and production and clearance of an estimated 10^12 ^virions per day in a given individual [63]. All currently recognized HCV genotypes are hepatotropic and pathogenic [64]. However, it has been suggested that different genotypes do vary in their infectivity and pathogenicity, thereby influencing the rate of progression to cirrhosis and the risk of HCC [65]. The duration of treatment should be based on the HCV genotype and the pretreatment viral load. Moreover, several distinct but closely related HCV sequences coexist within each infected individual.

There is 30-50% variation among viral genotypes and 15-30% among different subtypes while there is 1-5% variation in nucleotide sequence from a single HCV infected patient [66,67]. Genotype 1a and 1b is common in Western Europe. Genotype 3 is most frequent in the India, Nepal and Pakistan. Genotype 4 is the most common genotype in Africa and the Middle East. Genotype 5 is found in South Africa. Genotype 6 is found in Hong Kong and Southeast Asia [68]. Genotype 3a has a high prevalence worldwide, infecting up to 50% of patients in several European countries as well as a high percentage of HCV-infected individuals in many highly populated countries in Asia (eg. India). In Pakistan the major HCV genotype is 3a followed by 3b and 1a [69]. The prevalence rate was estimated to be 5.3% in Africa (31.9 million cases), 4.6% in the Eastern Mediterranean region (21.3 million cases), 3.9% in the West Pacific region (62.2 million cases), 2.15% in Southeast Asia (32.3 million cases), 1.7% in the Americas (13.1 million cases) and 1.03% in Europe (8.9 million cases) [70].

## Cell entry

**Figure 2:**
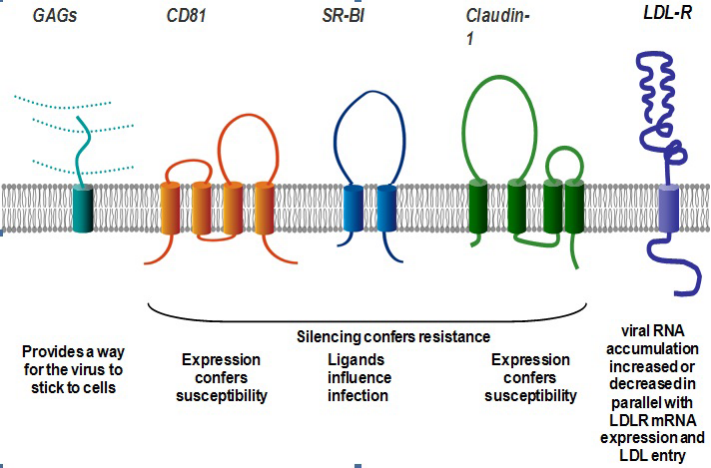
**HCV receptors for cell entry**.

Hepatitis C virus (HCV) entry is the first step of interactions between virus and the target cell that is required for initiation of infection. Recent studies suggest that HCV entry is a slow and complex multistep process. Several host cell surface molecules including glycosaminoglycans, CD81, scavenger receptor class B type I (SR-BI), members of the claudin family (CLDN1, 6 and 9) and mannose-binding lectins DC-SIGN and L-SIGN have been identified as putative HCV receptors or co-receptors (Figure 2) [71,72]. GAGs and the LDL-R may facilitate initial attachment to the host cell. This interaction is probably mediated by the lipoproteins associated with HCV virions. However, one cannot exclude direct contact between HCV envelope proteins and these cellular proteins. After the initial binding step, the particle likely interacts with SR-BI and CD81. HCV E2 binds with high affinity to the large external loop of CD81 and CLDN1 acts at a late stage of the entry process [71]. These receptors have been shown to play an important role for viral entry. Several human cell lines in spite off expressing all known entry factors still remain non-permissive the entrance of HCV. This finding suggests the requirement of some additional cellular factors that mediate entry of the virus. In recent years, several host restriction factors that protect cells from viral infection have been identified such as EW1-2wint [73,74]. EW1-2wint is a CD81 associated protein which is able to inhibit HCV entry into target cells by blocking the interactions between HCV glycoproteins and CD81. EWI-2wint may interfere with actin polymerization during viral entry or block signaling pathways necessary for viral entry [75].

## Viral RNA Transcription, replication and Translation

The virus linked to its receptor complex, internalize and then nucleocapsid is released into the cytoplasm. The virus is decapsidated, and the genomic HCV RNA is used both for polyprotein translation and replication in the cytoplasm. Being a positive sense RNA, viral RNA act as mRNA and is therefore directly translated. Translation of HCV RNA is not cap dependent like other cellular RNAs in which cap bind to ribosomal machinery for translation. Translation of HCV RNA is initiated by binding the 5^/^-IRES to ribosome. Translation of HCV RNA occurs at rough endoplasmic reticulum and produces single polyprotein which cleave by co and post-translationally by cellular and viral proteases, to produce structural and non structural proteins. Hepatitis C virus like other single stranded viruses of positive polarity induces alteration in membrane. These changes in the membrane termed as membraneous web [76,77]. NS5B RNA-dependent RNA polymerase replicates the genome by the synthesis of negative strand RNA. This negative strand RNA serves as a template for the synthesis of positive strand RNA. Replication and post-translational processing appear to take place in a membranous web made of the non-structural proteins and host cell proteins called "replication complex", located in close contact with perinuclear membranes. Genome encapsidation appears to take place in the endoplasmic reticulum and nucleocapsids are enveloped and matured into the Golgi apparatus before newly produced virions are released in the pericellular space by exocytosis [49].

## Treatment of HCV

HCV infection is rarely diagnosed during the acute phase. Therefore, the treatment of acute hepatitis is very limited. However, recent studies indicate that early treatment with interferon may be beneficial [78,79]. All patients with chronic HCV infection (defined as infection persisting over more than six months with positive HCV-antibody and HCV-RNA detectable in the serum) are candidates for therapy. In past, the treatment of standard is interferon alone or combination of interferon with ribavirin. Interferon α is a cytokine which plays an important role in innate immune responses towards viruses. The molecular mechanism by which IFN operates is mediated by its binding to receptors on the surface of the target cells, which lead to a phosphorylation cascade involving different tyrosine-kinases. Finally, transcription factors are recruited to the nucleus where they induce the synthesis of a number of antiviral effectors proteins [80]. Interferon alpha alone result in 16-20% sustained response after 12 months of treatment [81]. Ribavirin is a guanosine analog with a broad antiviral spectrum that enhances the sustained virological response obtained with IFN treatment. Ribavirin show antiviral effect by immune modulation of interferon signaling pathway, inhibition of inosine monophosphate dehydrogenase which results in GTP depletion. Ribavirin also has direct antiviral effect on RNA dependent RNA Polymerase and mutagenesis which results in reduction in virion infectivity [82]. Interferon in combination with ribavirin results sustained viral response in 35-40% of patients [83]. Special precautions should be taken before giving interferon to patients with decompensated cirrhosis, severe neutropenia, uncontrolled thyroid functions, thrombocytopenia, drug or alcohol abuse and past or current psychiatric illness.

Since the beginning of 2001, recombinant interferon has been replaced by newly developed pegylated IFN α2a and IFN α2b. The current standard therapy for hepatitis C treatment consists of combination of pegylated interferon α (Peg-IFN-α) with Ribavirin (RBV). This combination regimen is successful in patients with HCV genotypes 2 and 3 infection achieving HCV eradication rates of 75-90%. However, this combination is much less effective in patients with genotypes 1 and 4 infections with eradication rates ranging between 45% and 52% [84]. PegIFN α has an extended half-life compared to Interferon α and therefore can be administered only once a week. There are currently two pegIFN isoforms used for the treatment of chronic Hepatitis C virus, pegIFN2a (Pegassays, administered in a dose of 180 μg/week) and pegIFN2b (PegIntron, given in a dose of 1.5 μg/kg body weight). Pegasys is a 40 kDa molecule with a half-life of 60-80 h and PegIntron is a 15 kDa molecule with a half-life of 40 hours. Despite these differences in half-life, both drugs are injected once a week. The combination treatment can be administered to the relapse cases and people who do not respond to monotherapy. However, it remains difficult to treat patients co-infected with HIV or HBV, patients with high viremia titre, genotype 1 and 4, non-responders to monotherapy and patients with solid organ transplantation. The HCV genotype 3, younger age and female gender are significantly associated with high rate of spontaneous clearance of infection [85]. The best possible indicator of effective treatment is a sustained virological response (SVR), currently defined as undetectable HCV-RNA in peripheral blood determined with the most sensitive PCR technique 24 weeks after the end of treatment (ETR) [84].

As HCV infection causes increased oxidative stress, this can reduce the antiviral actions of interferon by blocking the c-Jun N-terminal Kinase/Signal Transducers and Activator of Transcription (JNK/STAT) pathway required for signalling by interferon. Therefore, despite the best available treatment for HCV, some patients do not respond to the therapy and relapse rate are very high even after the completion of full course of the treatment [86]. Cause for these differential responses to treatment is currently unknown. Other factors associated with non-response to treatment are high baseline viral load as well as high fibrosis stage, old age, male gender, African American race, obesity, alcohol intake and changes in the host immune response, e.g. high interleukin-8 (IL-8) and IL-10 serum levels [87]. The numbers of NS5A/ISDR mutations have also been shown to be relevant to the outcome of anti-HCV therapy [88]. Moreover, IFN-α is associated with serious adverse effects including leucopenia, thrombocytopenia, neutropenia, depression, fatigue, and "flu-like" symptoms. The addition of ribavirin, although enhancing the sustained viral response, is also associated with a serious side effect like hemolytic anemia. These side effects are sometimes dose limiting and may lead to discontinuation of treatment [89]. These side effects result in discontinuation of therapy in 20% of patients [90]. Because of extreme sequence variability within the HCV genome, development of an effective vaccine against HCV infection has proven to be very difficult. Furthermore, combination therapy is expensive. There is therefore a great need to develop new therapeutic approaches that effectively block the different HCV genotypes and avoid the appearance of resistant quasispecies.

## Immune responses in HCV infection

Innate and adaptive immune responses play an important role in viral infection. In the liver the innate immune responses are due to NK cells, NKT cells, kupffer (liver macrophages) and a rapid interferon response exerted by infected liver cells. NK and NKT cells perform cytotoxic lysis of infected cells by releasing granules containing perforin and proteases (granzymes). NK and NKT cells also produce large amount of type II interferon gamma and TNF alpha. Production of double stranded RNA intermediates during HCV replication result in the activation of type I interferon gene. The host cells recognize double straded RNA via Toll like receptor 3 (TLR-3) [91]. The activation of type I interferon, interferon α and interferon β, play an important role in early defense system of viral infection. One study showed that the mice lacking interferon α and interferon β failed to clear HCV infection [92]. Similarly, humans with genetic defects in STAT-1, which is involved in the signaling cascade of the IFN system, die of viral disease at an early age (Dupuis et al., 2003). Another study in chimpanzees showed that interferon type 1 induced double-stranded RNA-dependent protein kinase (PKR), 2'-5' oligoadenylate synthetase (OAS) and Mx genes upon acute HCV infection, which play an important role to inhibit replication of HCV and induce apoptosis in infected hepatocytes [93]. Natural killer cells play an important role and can eliminate virus without any detectable HCV specific T cells responses in chimpanzee [94].

Adaptive immune response consist of humoral immune response such as antibody producing B cells and cellular immune responses such as CD+4 T helper cells (TH) and CD8+ cytotoxic T lymphocytes (CTLs). Virus-specific antibodies are usually detectable approximately 7-8 weeks after HCV infection [95]. It is still no completely underdtood whether or not these antibodies neutralize HCV infectivity. HCV infection neutralize in vitro with the treatment of antibodies [96] but naturally acquired antibodies fail to neutralize HCV infection in chimpanzee and human. Now, important progress has been made by the production of infectious lentiviral pseudotype particles bearing native HCV envelope glycoproteins. These pseudoparticles have been used to show cross-viral genotype neutralization of HCV by serum antibodies from chronically infected subjects [13,97]. CD+4 T helper cells (TH) and CD8+ cytotoxic T lymphocytes (CTLs) play an important role in virus particle clearance [98,99]. CD4+ T cells recognize antigens presented by MHC class II molecules on the surface of professional antigen presenting cells (APCs). CD4+ T cells perform multiple effector functions, including direct activation of macrophages and antigen-specific B cells as well as activation of CD8+ T cells. CD8+ T cells recognize antigens presented by MHC class I molecules on the surface of infected cells. CD8+ T cells perform different effector functions, such as the killing of infected target cells and the secretion of cytokines such as IFN gamma and TNF α that can inhibit viral replication without killing the infected cell [100]. However, detection of CD4+ and CD8+ T cell responses during acute phase of infection is an important prediction of outcome of infection.

## Viral evasion strategy to HCV

HCV interferes with innate and adaptive immune responses in several ways. HCV impair the interferon signaling pathway and HCV NS3/4A block RIG-1 activation and translation of IRF-3. HCV core protein interferes with the JAK/STAT pathway by activating the JAK-STAT signaling inhibitor SOCS-3 [101], increases the degradation of STAT1 and inhibits activation and translocation of STAT1 [89,102]. HCV E2 inhibit protein kinase receptor (PKR) activity and natural killer cell function [103,104]. HCV NS5A also plays an important role in escape of antiviral action of interferon. NS5A consists of 40 amino acid sequence termed as interferon sensitive region (ISDR) which plays an important role in responsiveness of interferon therapy. NS5A also interferes with the 2-5 OAS/RNaseL pathway by binding to 2-5 OAS [105]. Moreover, NS5A was shown to directly bind to protein kinase receptor ( PKR) and down-regulate the PKR expreswsion [106]. NS5A induces IL-8, a chemokine which inhibits the antiviral actions of IFN [107]. Envelope protein E2 crosslinks the HCV receptor CD81, thereby inhibiting cytotoxicity and IFN production by NK cells [103,104].

HCV escape from adaptive immune response through several different mechanisms such as mutational escape and functional anergy (failure) of virus specific T cells. Cellular immune responses such as CD4+ T helper cells (TH) and CD8+ cytotoxic T lymphocytes (CTLs) cause long lasting inflammatory reactions resulting in liver cirrhosis and hepatocellular carcinoma [108]. Weak T cell responses resulted poor controlled viremia and persistence [98]. In those patients that have been chronically infected, HCV specific CD8+ T cells may partially control viral replication as well as contributing to progressive liver disease. Mutational escape of HCV virus from the adaptive immune response has one of the major viral evasion strategies. Mutational changes in virus particle are due to lack of proofreading activity of RNA dependent RNA polymerase and high replication rate such as 10^12 ^virions per day. Sequence changes in the hypervariable region of the E2 envelope glycoprotein result in escape from B cell epitopes. Viral amino acid substitutions that inhibit HCV-specific T cell recognition have initially been observed in chronically HCV infected patients [109,110] and chimpanzees [111]. HCV mutations affect virus specific CD8+ T cell responses by decreasing binding affinity between epitope and MHC molecule by decreasing T cell receptor (TCR) recognition [112] and impairing proteosomal processing of HCV antigens[113].

Another important possible mechanism of immune evasion is functional anergy of virus-specific T cells. Several studies have shown that dysfunction of CD8+ T cells occurs in acute as well as chronic HCV infection [98,114]. HCV-specific CD8+ T cells may be impaired in their proliferative capacity, cytotoxicity, and ability to secrete TNF α and IFN λ [115].

## Conclusion

HCV infection is a serious global health problem necessitating effective treatment. Presently, there is no vaccine available for prevention of HCV infection due to high degree of strain variation. Current therapeutic options for hepatitis C are limited, especially for genotype 1. For genotypes 2 and 3, pegylated interferon in combination with ribavirin, can lead to a sustained virological response in up to 80% of patients [116]. However, the therapy is expensive and often associated with side effects that may lead to discontinuation of therapy. In this review, we summarize molecular virology, replication and immune responses against HCV and discussed how HCV escape from adaptive and humoral immune reseponses. Recent advances in our understanding of HCV structure, genome lifecycle and immune responses have revealed numerous target sites for potential pharmacological intervention. These should help in further improving HCV treatment and development of vaccine against HCV.

## Competing interests

The authors declare that they have no competing interests.

## Authors' contributions

UAA designed the study, analyze the data and wrote paper. All authors read and approved the final manuscript.
